# Correlation between change in muscle excursion and collagen content after tendon rupture and delayed repair

**DOI:** 10.1186/s13018-017-0518-y

**Published:** 2017-01-23

**Authors:** Il-Hyun Koh, Ho-Jung Kang, Won-Taek Oh, Jung-Jun Hong, Yun-Rak Choi

**Affiliations:** Department of Orthopaedic Surgery, Yonsei University College of Medicine, 50-1 Yonsei-ro, Seodaemun-gu, Seoul, 120-752 Republic of Korea

**Keywords:** Collagen subtypes, Muscle excursion, Tendon rupture, Delayed tendon repair

## Abstract

**Background:**

The objectives of the present study were to compare changes in muscle excursion, total collagen, and collagen subtypes after tenotomy over time and after delayed tendon repair.

**Methods:**

Tenotomy on the extensor digitorum tendon of the right second toes of 48 New Zealand White rabbits was performed; toes on the left leg were used as controls. Passive muscle excursion, total collagen content, and type I, III, and IV collagen contents were measured at 1, 2, 4, and 6 weeks after tenotomy. Next, passive muscle excursion and total collagen content were measured at 8 weeks after delayed tendon repair at 1, 2, 4, and 6 weeks after a tenotomy.

**Results:**

Passive muscle excursion decreased sequentially over time after tenotomy. Meanwhile, total collagen increased over time. These changes were significant after 4 weeks of injury. Type I collagen significantly increased, type III collagen significantly decreased, and type IV collagen had no significant change over time. Passive muscle excursion was negatively correlated with total collagen and type I collagen after tenotomy at each time point after tenotomy (*p* < 0.05). After tendon repair, increases in total collagen content after tenotomy were not reversed, despite early repairs at 1 and 2 weeks after tenotomy.

**Conclusions:**

Increases in type I collagen were found to be associated with decreased excursion after tendon rupture. The increase in collagen that was observed after tenotomy was not reversed by repair within 8 weeks.

## Background

Tendon injuries are often overlooked and untreated. Left alone, these injuries eventually reduce active joint motion [1]. Prolonged muscle retraction after tendon rupture causes intracellular changes, including Z band streaming, shortened sarcomere lengths, central core degenerative changes, changes in the myosin heavy chains, titin isoform changes, and decreases in sarcomere number, as well as extracellular changes [2–5]. The latter changes include an increase in collagen and reduced numbers of capillaries [6]. In pathologic muscle, increased collagen content is believed to be an important factor in excursion reduction [7].

Collagen is an important component of the extracellular matrix (ECM) of connective tissue layers that surround muscle cells [8]. We reported that muscle excursion was decreased and collagen content was increased over time after tenotomy and that collagen content was negatively correlated with passive excursion [9]. Type I, III, and IV collagen are the main subtypes of muscle collagen [10, 11]. It is thought that type I collagen resists tension and type III collagen provides structural stability to expandable organs [12]. Though total collagen content has been shown to increase over time after tenotomy, changes in collagen subtypes over time after tenotomy have yet to be described. We propose that changes in collagen subtypes over time after tenotomy would help our understanding of decreases in muscle excursion and provide therapeutic clues for neglected tendon ruptures.

It is unknown if delayed repair after tenotomy can reverse decreased muscle excursion and increased collagen content over time after tenotomy or when the delayed repair effect disappears after tenotomy. This information is an important clinical decision-making tool when choosing surgical methods, such as direct repair, tendon transfer, or tendon graft, for treatment of a neglected tendon rupture. Accordingly, this study was designed to examine whether collagen subtypes in a tenotomized muscle change over time and whether changes in total collagen content and muscle excursion after tendon rupture are reversed by delayed tendon repair.

## Methods

### Materials

All experimental methods were approved by the Institutional Animal Care and Use Committee of Yonsei University. New Zealand White rabbits (mean body mass, 3.1 ± 0.5 kg; *n* = 48 subjects, 96 hind limbs) were obtained from DooYeol Biotech (Seoul, South Korea). Rabbits were cared for in accordance with the guidelines of the NIH Guide for the Care and Use of Laboratory Animals. Rabbits were single-housed in standard stainless steel cages and allowed free access to standard rabbit chow and water, with 12-h photoperiod (light on at 8:00, light off at 20:00), 17–21 °C ambient temperature, and 50 ± 10% relative humidity.

### Experimental design

The extensor digitorum muscle of the second toe (EDII) was chosen as the experimental muscle because it has a long and large tendon that can be easily manipulated surgically. This muscle is easy to define because all muscle fibers of the muscle of the second toe originate from the medial surface of the tibia. The EDII tendon is also an intrasynovial tendon, as are the human finger flexors, which are commonly injured [13].

Animals were randomly assigned to eight treatment groups (six rabbits in each) at the beginning of the study. For the first experiment, tenotomy of the EDII was performed on the right legs and the intact left legs served as a control. Passive muscle excursion and the content of hydroxyproline, as well as type I, III, and IV collagen, were measured at 1, 2, 4, or 6 weeks after tenotomy. For the second experiment, tenotomy of the EDII was performed on the right legs and the intact left legs served as the control. The tendon was repaired at 1, 2, 4, or 6 weeks after tenotomy. Passive muscle excursion and hydroxyproline content were measured in both legs at 8 weeks after repair.

### Surgical procedures and measurements

#### Tenotomy

Anesthesia was induced with an intramuscular injection of 15 mg/kg of Zoletil 50 (Zoletil 50 mg/mL, [tiletamine 125 mg, zolazepam 125 mg]; Virbac, Carros, France) and 5 mg/kg of Rompun (xylazine hydrochloride 23.32 mg/mL; Bayer Korea, Seoul, South Korea) and was maintained with enflurane, which provided approximately 30 min of adequate sedation. The right hind limbs of the animals were shaved and positioned supine on the operating table. The knee was in approximately 90° of flexion and the hip was in flexion and external rotation. Under aseptic conditions, a longitudinal skin incision was made over the medial side of the distal tibia and the tendon of the EDII was exposed. The tendon was transected at the level of the metatarsal. The wound was closed and animals were returned to their cages.

#### Tendon repair

At various times after the tenotomy was performed as described above, animals were anesthetized, their incisions were re-opened, and the proximal tendon stump of the muscle was completely released from the adhesions with the surrounding tissues. Because end-to-end repair was impossible due to the decreased muscle excursion, the tendon was sutured to the ankle extensor retinaculum at the point of 50% excursion. After wound closure, animals were allowed to move freely in their cages.

#### Measurement of excursion

The proximal tendon stump of the muscle was completely released from the adhesions with the surrounding tissues before the measurement of muscle excursion. A marking suture was placed in a proximal tendon end in the myotendinous junction, which was selected as a measurement point to avoid tendon length change through tendon stretching. The EDII tendon was pulled with a hemostat by the operating surgeon. Traction force was maximally applied to the EDII tendon to the extent that further plastic deformation did not occur by stretching of the tendon. The location of the marking suture in the retracted position and the maximal manual traction position were marked in the medial tibia bone. Muscle excursion was obtained by measuring the distance between the two bone markers using a digital caliper.

#### Measurement of hydroxyproline in EDII

Animals were sacrificed with an overdose of pentobarbital (150 mg/kg i.p.). The EDII were harvested from the bilateral lower limbs after measuring muscle excursion. Hydroxyproline, a major component of collagen, was quantified as a measure of collagen content using the Hydroxyproline Colorimetric Assay Kit (BioVision, San Francisco, CA, USA) according to the manufacturer’s instructions.

#### Measurement of type I, III, and IV collagen content in EDII

Collagen was solubilized with pepsin under acidic conditions and then further digested with pancreatic elastase at neutral pH to convert polymeric collagen to monomeric collagen. Type I, III, and IV collagen were measured using specific enzyme-linked immunosorbent assay (ELISA) kits (www.antibodies-online.com; catalog numbers: ABIN628250, ABIN628251, and ABIN628256, respectively) according to the manufacturer’s instructions.

#### Statistical analysis

SPSS version 20 (SPSS Inc., Chicago, IL, USA) was used for all statistical analyses. The muscle excursion, the content of hydroxyproline, and type I, III, and IV collagen content relative to controls were used to account for individual differences between rabbits. The values for these parameters at each time point after tenotomy were compared in a linear mixed model. Muscle excursion and hydroxyproline content relative to control values at 8 weeks after tendon repair were compared with a linear mixed model, according to repair timing. The muscle excursion ratios and the hydroxyproline ratios to the control value after tenotomy and after delayed tendon repair were compared using the Mann-Whitney test according to the period after the tenotomy. Correlations between muscle excursion ratio in relation to the control value and hydroxyproline and type I, III, and IV collagen content ratios to control values at each time point after tenotomy and between the muscle excursion ratio to the control value and hydroxyproline content ratio to the control value at 8 weeks after tendon repair were also analyzed. For all analyses, the level of significance was *p* < 0.01.

## Results

All eight treatment groups of six rabbits survived without complications until scheduled sacrifice.

### Passive muscle excursion after tenotomy

**Fig. 1:**
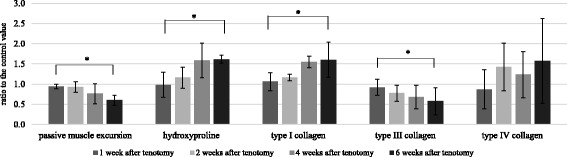
Passive muscle excursion and collagen content of the EDII muscle of rabbits at 1, 2, 4, or 6 weeks after tenotomy. Data represent means ± SE and are relative to the control values. *Significant difference between groups (*p* < 0.01)

The mean passive muscle excursions after tenotomy decreased from 3.7 ± 0.5 mm at 1 week to 2.4 ± 0.4 mm at 6 weeks. The mean ratios thereof in relation to control values significantly decreased over the same time period from 94 ± 6 to 60 ± 12% (*p* < 0.01; Table 1, Fig. 1).

**Table 1:** Parameters of EDII muscles of rabbits at various times after tenotomy

Time after tenotomy		1 week	2 weeks	4 weeks	6 weeks	*p* value
Passive muscle excursion	mm	3.7 ± 0.5	3.7 ± 0.3	3.1 ± 0.8	2.4 ± 0.4	
	Ratio	0.94 ± 0.06	0.92 ± 0.13	0.76 ± 0.25	0.60 ± 0.12	<0.001
Hydroxyproline content	ng/mg	591.86 ± 82.52	678.95 ± 94.93	865.21 ± 171.95	740.09 ± 240.86	
	Ratio	0.98 ± 0.31	1.16 ± 0.27	1.59 ± 0.43	1.62 ± 0.10	<0.001
Type I collagen content	ng/mg	32.85 ± 5.94	49.88 ± 1.59	64.88 ± 2.53	65.39 ± 9.81	
	Ratio	1.06 ± 0.22	1.17 ± 0.08	1.55 ± 0.14	1.61 ± 0.43	<0.001
Type III collagen content	ng/mg	9.03 ± 1.91	7.34 ± 1.96	6.58 ± 1.48	5.62 ± 1.68	
	Ratio	0.92 ± 0.20	0.78 ± 0.20	0.68 ± 0.29	0.57 ± 0.33	<0.001
Type IV collagen content	ng/mg	1.89 ± 1.62	2.27 ± 1.01	2.68 ± 0.55	2.70 ± 1.79	
	Ratio	0.87 ± 0.48	1.43 ± 0.59	1.24 ± 0.57	1.57 ± 1.05	0.106

Data are expressed as means ± SE and relative to the control values

### Hydroxyproline content of muscle after tenotomy

**Fig. 2:**
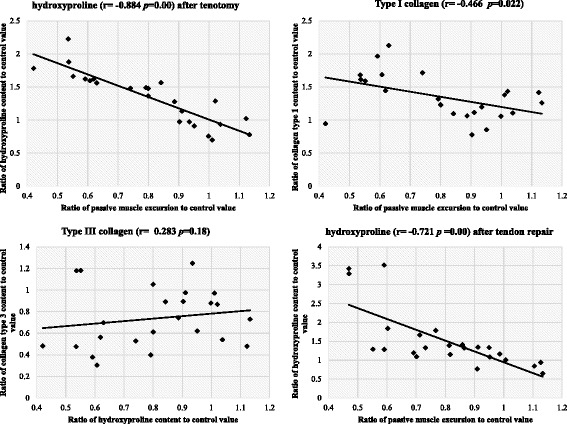
Correlations between excursion ratios relative to the control values for several parameters in EDII muscles of rabbits after tenotomy and after tenotomy with delayed tendon repair

Hydroxyproline content in the muscle was measured in order to quantify collagen content. Hydroxyproline content in the muscle after tenotomy increased from 591.86 ± 82.52 ng/mg at 1 week to 740.09 ± 240.86 ng/mg at 6 weeks. The mean ratio of hydroxyproline content in muscle after tenotomy to the control values significantly increased from 98 ± 31% at 1 week to 162 ± 10% at 6 weeks (*p* < 0.01; Table 1, Fig. 1). We observed a significant correlation between muscle excursion values and hydroxyproline content after tenotomy (*r* = −0.884, *p* = 0.00; Fig. 2).

### Collagen content of muscle after tenotomy

Type I collagen content in the muscle after tenotomy increased from 32.85 ± 5.94 ng/mg at 1 week to 65.39 ± 9.81 ng/mg at 6 weeks. The mean ratio of type I collagen content in muscle after tenotomy to the control value significantly increased from 106 ± 22% at 1 week to 161 ± 43% at 6 weeks (*p* < 0.01; Table 1, Fig. 1).

Type III collagen content in the muscle after tenotomy decreased from 9.03 ± 1.91 ng/mg at 1 week to 5.62 ± 1.68 ng/mg at 6 weeks. The mean ratio of type III collagen content in muscle after tenotomy to the control value significantly decreased from 92 ± 20% at 1 week to 57 ± 33% at 6 weeks (*p* < 0.01; Table 1, Fig. 1).

Type IV collagen content in the muscle after tenotomy increased from 1.89 ± 1.62 ng/mg at 1 week to 2.70 ± 1.79 ng/mg at 6 weeks. The mean ratio of type IV collagen content in muscle after tenotomy to the control value exhibited an increasing trend, although it was not statistically significant (*p* = 0.106; Table 1, Fig. 1).

There was also a significant correlation between muscle excursion values and the amount of type I collagen after tenotomy (*r* = −0.466, *p* = 0.022). There was no significant correlation between muscle excursion values and type III collagen content (*r* = 0.283, *p* = 0.18) or type IV collagen content (*r* = −0.19, *p* = 0.373) after tenotomy (Fig. 2).

### Passive muscle excursion after tendon repair

**Fig. 3:**
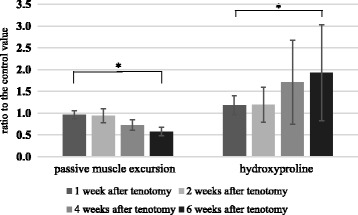
Passive muscle excursion and hydroxyproline content as a measure of total collagen after delayed tendon repair in the EDII muscle of rabbit. Data are expressed as means ± SE relative to the control values. *Significant difference between groups (*p* < 0.01)

**Fig. 4:**
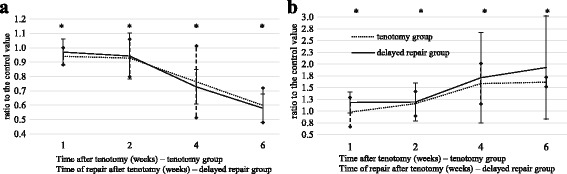
Passive muscle excursion (**a**) and hydroxyproline content (**b**) in the EDII muscle of rabbits at different times after tenotomy. The tendon was repaired at the indicated time in the delayed repair group. Data are expressed as means ± SE and relative to the control values. *Significant difference between groups (*p* < 0.01)

Mean passive muscle excursions after tendon repair according to timing of delayed repair significantly decreased over time (*p* < 0.01). The mean ratios of muscle excursion according to timing of delayed repair to control values significantly decreased from 97 ± 9% at 1 week to 58 ± 10% at 6 weeks (*p* < 0.01; Table 2, Fig. 3). Significant differences in excursion values relative to the control were observed between tenotomy and delayed tendon repair groups at all time points (*p* < 0.01; Fig 4).

**Table 2:** Passive muscle excursion and hydroxyproline content of the EDII muscle of rabbit after tenotomy with delayed tendon repair

Time after tenotomy		1 week	2 weeks	4 weeks	6 weeks	*p* value
Passive muscle excursion	mm	3.7 ± 0.5	3.8 ± 0.5	3.0 ± 0.5	2.4 ± 0.3	
	Ratio	0.97 ± 0.09	0.94 ± 0.16	0.73 ± 0.12	0.58 ± 0.10	<0.001
Hydroxyproline content	ng/mg	644.27 ± 64.11	684.24 ± 187.93	791.42 ± 152.78	792.10 ± 311.20	
	Ratio	1.18 ± 0.22	1.20 ± 0.40	1.71 ± 0.96	1.93 ± 1.10	<0.001

Hydroxyproline was used as a measure of total collagen. Data are expressed as means ± SE and relative to the control values

### Hydroxyproline content as a measure of total collagen in muscle after tendon repair

Hydroxyproline content in the muscle after tendon repair increased with the timing of the delayed repair. The mean ratios of hydroxyproline in muscle after delayed tendon repair to control values significantly increased over time (*p* < 0.01; Table 2, Fig. 3). Significant differences in hydroxyproline content relative to control were observed between tenotomy and the delayed tendon repair groups according to time after tenotomy (*p* < 0.01; Fig. 4). We noted a significant correlation between the muscle excursion values after tendon repair and the hydroxyproline content after delayed tendon repair (*r* = −0.721, *p* = 0.00, Fig. 2).

## Discussion

To delineate the relationships between changes in muscle excursion and collagen content after tendon rupture and delayed repair, tenotomy on EDII muscles of rabbits was performed on the right legs with or without subsequent repair. Intact left legs served as controls. Muscle excursion, hydroxyproline content, and type I, III, and IV collagen content relative to control were used to account for differences between individuals. Total collagen and type I collagen significantly increased with time after tenotomy, excursion and type III collagen decreased significantly, and there was no significant change in type IV collagen. Muscle excursion ratio was significantly negatively correlated with total collagen and type I collagen after tenotomy. Total collagen content at 8 weeks after tendon repair increased significantly, and muscle excursion decreased significantly with delay in repair, such that excursion was significantly negatively correlated with total collagen. The increase in collagen that was observed after tenotomy was not reversed by repair. Improvement in excursion was noted when repair was delayed by 1 or 2 weeks; however, when it was delayed by 4 or 6 weeks, excursion decreased. Increases in type I collagen were associated with decreased excursion after tendon rupture.

In the present study, the ratio of type I to type III collagen at 1 week after tenotomy was 3.6:1. Type I collagen was negatively correlated with passive excursion, while type III was positively correlated with passive excursion. Type I and III collagen are important components of the perimysium and epimysium, and their relative amounts differ between species and between muscle groups within the same species [10, 11]. Therefore, different ratios might be observed between type I and type III collagen content if excursion is analyzed according to hydroxyproline content. Type I collagen, a fibrillar collagen, is composed of a bundle of thick fibrils with an average diameter of 75 nm. It is thought to resist tension and to decrease excursion via increased passive stiffness. In contrast, type III collagen is composed of loosely packed bunches of thin fibers with a diameter of 45 nm and provides structural stability to expandable organs, such as the uterus [12]. The difference in tensile stress between type I and type III collagen may determine passive stiffness and excursion. If there is a high ratio of type I to type III collagen in a muscle, increased passive stiffness and decreased excursion would be expected. Therefore, measures that suppress increases in type I collagen content might prevent a decrease in excursion after tendon rupture.

Type IV collagen is a non-fibrillar collagen and an important component of the endomysium [14]. It forms a reticular meshwork of individual muscle fibers and may function in resistance to multi-directional, rather than longitudinal, tension [15]. In addition, the endomysium connects individual muscle fibers through a basement membrane and is important in lateral force transmission [16]. In the present study, type IV collagen content relative to control tended to increase over time after tenotomy. One study reported that, after high-force eccentric contraction, endomysium thickness increased as type IV collagen contents of the skeletal muscle increased [17]. Furthermore, because additional type IV collagen outside the ECM is distributed widely in the basal lamina of microvessels and vessel density decreases after tenotomy, changes in microvessels may be an important factor when analyzing type IV collagen content [18]. Another report stated that the synthesis of type I, III, and IV collagen in muscles decreases after long periods of immobilization [19]. These results are confounding factors in the interpretation of our results. However, an increase in type IV collagen content may be related to thickening of the endomysium, as the amount of type IV collagen tended to increase relative to control over time.

Passive muscle excursion decreased sequentially with time after tenotomy, becoming prominent at 4 and 6 weeks after tenotomy. The decreased excursion at 4 and 6 weeks after tenotomy did not increase after delayed repair. The hydroxyproline content relative to control increased after repair (Fig. 4). Therefore, the accumulation of collagen in response to tenotomy was not reversed within the 8 weeks of this study. In particular, a gradual increase in hydroxyproline content was observed at 4 and 6 weeks in the delayed tendon repair group. This is considered to be caused by fibrosis resulting from microcirculation dysfunction of fixed muscle in an overstretched state, which is induced via a decrease in excursion over time. The increased collagen content may limit the degree of tendon repair, causing pathologic positive feedback of fibrosis and preventing improvement in excursion.

There are several limitations to the present study. First, the applied maximal passive tension may be subjective when measuring passive excursion. Muscle excursion has been measured during full flexion and extension of joints using ultrasonography or magnetic resonance imaging, but in cases of tendon rupture, excursion measurement by joint motion is impossible [20, 21]. In this study, excursion was measured by manual traction to the extent that further plastic deformation did not occur. Tendon traction using a specific weight was thought to be a more objective method than manual traction; however, because excursion could vary according to the weight applied to a tendon, it would be difficult to determine the proper weight and interpret the results. Although maximal manual traction force might be subjective, this measurement method was thought to be more applicable and reproducible in a clinical situation [22]. Second, our study focused on the relationship between passive muscle excursion and collagen content following tenotomy and delayed tendon repair. Sarcomere number also is considered to be an important factor in muscle excursion, and a change in sarcomere number after tenotomy with delayed repair could be important in muscle excursion. Understanding the effect of the amount of collagen on muscle excursion will require assessing the effect of a decreased sarcomere number in passive excursion. Third, instead of end-to-end repair, the tendon was sutured to the ankle extensor retinaculum at the point of 50% excursion. We thought that muscle shortening and tendon degeneration due to neglected tendon rupture would make it difficult to perform end-to-end repair and excessive stretching could lead to muscle fibrosis. In experiments with rabbit soleus muscle, normal recovery of electrophysiological properties was reported after 8 weeks in a tendon lengthening group maintaining half of the excursion [22]. In the case of tenodesis by overstretching rabbit EDII tendon, reduction of muscle excursion, increase in number of sarcomeres, and increase in passive tension were reported [13]. Since the method used in this study could not maintain excursion, we were unable to compare our results with the end-to-end repair method. In order to confirm this difference, additional studies maintaining excursion without excessive lengthening, such as end-to-end repair using a tendon lengthening method or tendon transfer to the proximal tendon, are needed.

## Conclusions

Increases in type I collagen were found to be associated with decreased excursion after tendon rupture. The increase in collagen that was observed after tenotomy was not reversed by repair within 8 weeks.

## Abbreviations

ECM: Extracellular matrix
EDII: Extensor digitorum muscle of the second toe
